# Fabrication and Actuation of an Electrowetting Droplet Array on a Flexible Substrate

**DOI:** 10.3390/mi8110334

**Published:** 2017-11-18

**Authors:** Kari L. Van Grinsven, Alireza Ousati Ashtiani, Hongrui Jiang

**Affiliations:** Department of Electrical and Computer Engineering, University of Wisconsin-Madison, Madison, WI 53706, USA; kvangrinsven@wisc.edu (K.L.V.G.); ousatiashtia@wisc.edu (A.O.A.)

**Keywords:** electrowetting actuators, flexible materials, electrowetting array, EWOD

## Abstract

Electrowetting-on-dielectric (EWOD) is a fast, well-established actuation method for a variety of applications, from microfluidics to electrowetting displays to electrowetting lenses. We therefore seek to develop a robust, scalable fabrication method for the realization of EWOD on a flexible polydimethylsiloxane (PDMS) substrate in order to increase the range of possible applications. We fabricated a 5 × 5 array of individually controlled electrowetting cells to manipulate silicone oil droplets via EWOD. The fabrication process utilized exclusively flexible materials to improve the robustness of the overall device, and processing methods were adapted to accommodate the particular challenges posed by flexible materials. Simulation of the EWOD devices was conducted using ANSYS Fluent and showed the change in contact angle in response to voltage applied. Fabricated devices were also tested, with actuation of the oil droplet observed with up to 100 V (RMS) AC applied across underlying electrodes. We demonstrated fabrication of a fully flexible array and verified actuation to center droplets over the electrodes. This work may be expanded to address more specific flexible applications for EWOD.

## 1. Introduction

### 1.1. Background

The use of microfluidic systems for precise manipulation and analysis of liquids has long been established as a crucial tool for a large array of fields, including biological processes such as cell sorting and counting in particular [1]. While microfluidic systems take advantage of a large array of actuation methods—including pressure, capillary, pumps, and various electrokinetic mechanisms—few provide the degree of reconfigurability demonstrated by electrowetting-on-dielectric (EWOD) lab on chip systems. In such systems, transport of droplets does not require a physical, predefined microchannel but instead a droplet can be induced to move along a reconfigurable path in a plane by applying voltage potentials across underlying electrodes insulated from the liquid itself via a dielectric layer [2,3,4,5]. These electrowetting based systems have also been shown to perform a large variety of microfluidic tasks beyond mere transport, including droplet creation, merging/mixing of droplets, cutting/dividing of droplets, and liquid lens actuation [4,6]. More complicated systems which utilize EWOD actuation in combination with dielectrophoresis (another electrohydrodynamic actuation method) have been used for manipulating cell concentrations within fluid droplets [7]. However, none of these EWOD microfluidic systems has been extended to fabrication on a flexible substrate. Substrate flexibility allows for a wider range of applications for the system. For instance, it is well known that substrate flexibility is a regulatory parameter for certain kinds of cell growth [8]. Integration of cell growth, transport, and measurement into a single system could therefore benefit from the use of a single flexible substrate for all functions.

Alternative situations where flexibility of the underlying substrate may prove useful is in electrowetting displays, which utilize EWOD to manipulate black or colored liquid droplets in individual cells to act as pixels on a display by being actuated from on to off state by applied voltage. As interest grows in flexible and wearable electronics, flexible electrowetting displays may become useful [9]. Wearable microfluidic systems also benefit from use of electrowetting as actuation method, since electrowetting allows for simpler interconnects for modular wearable devices [10]. Finally, liquid lenses with focal lengths tunable via electrowetting or dielectrophoresis may prove particularly useful in applications such as soft contact lenses capable of correcting presbyopia. In addition, there are a number of examples of actuation of lenses on flexible surfaces [6,11], but combining these lenses into an array has not been deeply explored. An array of tunable lenses on a flexible substrate would allow for a reconfigurable imaging system. An array of individual electrowetting cells is also useful in the case of electrowetting optical switches [12,13].

All of these applications motivate us to derive a robust, scalable EWOD system fabricated on flexible substrates. The particular electrode design chosen focuses on the manipulation of an oil droplet in an aqueous solution to vary the surface area covered (i.e., contact angle) and center the oil droplet over the electrode; however, the fabrication method we provide here could be easily adapted to a large variety of other electrowetting applications by utilizing different electrode geometries, or aqueous liquids could be actuated on the same electrode structure using dielectrophoresis. In our configuration, the electrodes are laid out in a 5 × 5 array which form the basis for a 5 × 5 array of individual electrowetting cells once assembly is complete. Each electrowetting cell can be individually actuated via its own voltage control, and the entire array is fabricated on a single, flexible polydimethylsiloxane (PDMS) substrate. 

### 1.2. Concept and Acutation Mechanism

**Figure 1:**
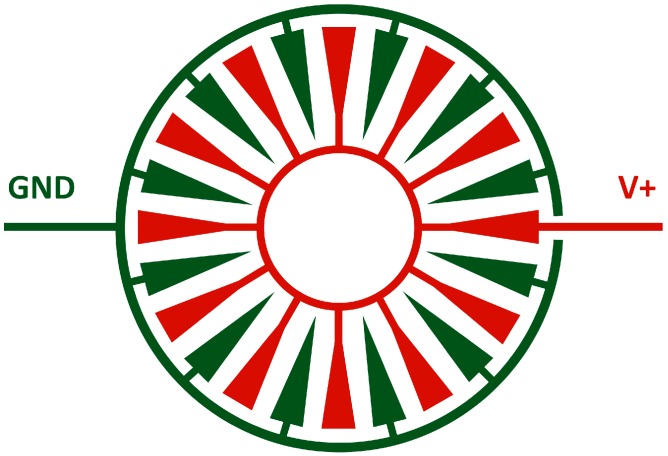
Schematic of areal density modulated electrodes used for electrowetting actuation. The interdigitated electrodes (+V) and counter electrodes (GND) cover an increasing percentage of the available area as the radius from center increases. This ensures that all forces on the oil droplet will be symmetric and that the droplet will be centered over the electrodes as voltage is applied.

Each electrowetting cell in the array is composed of areal density modulated electrodes (shown in Figure 1) which we previously demonstrated provided good electrowetting actuation [14], but which have now been patterned on a flexible PDMS substrate. The electrodes are insulated by a dielectric layer with a patterned hydrophobic region over the electrodes as well as a PDMS ring to create a well which contains the two immiscible liquids actuated by the electrowetting. A droplet of silicone oil, an insulator, is placed atop the underlying electrowetting electrodes and then surrounded by water which is conductive and serves as a floating electrode. As voltage is applied across the underlying areal density modulated electrodes, the liquids in the electrowetting cell will shift to minimize the potential energy of the system, since minimizing the potential energy is equivalent to maximizing the capacitance of the system according to Equation (1):

(1) $$U=\frac{1}{2}\frac{q^{2}}{C}\;,$$

where U is the potential energy of the system, q is the charge, and C is the capacitance. By design, the potential energy can only be at a minimum when the silicone oil droplet is centered over the areal density modulated electrodes [14]. As the voltage across the underlying electrodes is increased, the conductive water, which functions as a floating electrode, is attracted down toward the electrodes to minimize the energy in the system by minimizing the distance between itself and the underlying electrodes. This means that the water will seek to cover more and more of the surface immediately over the electrodes as the voltage increases, effectively squeezing the silicone oil droplet which it surrounds.

By allowing the conductive liquid to act as a floating electrode instead of applying voltage directly across the conductive liquid, we choose to further enhance the robustness of the electrowetting application at the cost of increasing the voltage required to induce actuation. The robustness is further enhanced by applying an AC voltage to the system in order to reduce the chances of dielectric breakdown in the device.

## 2. Materials and Methods

**Figure 2:**
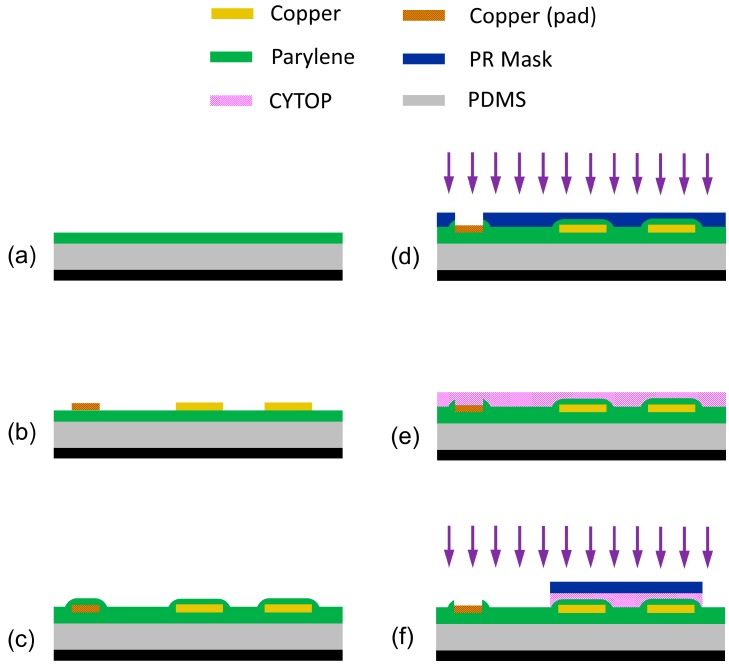
Micromachining fabrication process of flexible electrowetting array. (**a**) 250 µm of PDMS was spin coated onto a carrier wafer. After PDMS was cured a 10 µm layer of Parylene C was deposited as an adhesive layer; (**b**) 275 nm of copper was deposited via magnetron sputtering and then patterned and wet etched into areal density modulated array electrodes along with traces and pads; (**c**) Another 2.5 µm of Parylene C was deposited over the whole wafer to act as a pinhole free dielectric layer; (**d**) The Parylene C insulator was etched in the region directly over pads for electrical contacts. A thick photoresist was patterned to act as a mask while Parylene C was etched used 400 W oxygen plasma; (**e**) 1 µm of CYTOP was spin coated and cured to create a highly hydrophobic surface. The curing was done at a low temperature to minimize thermal strain to the flexible substrate; (**f**) CYTOP layer was pattered using a photoresist mask to protect the disk directly over the electrowetting electrodes. CYTOP was etched using 200 W oxygen plasma.

From start to finish, the conception and execution of the flexible substrate for our EWOD array required careful consideration of materials and fabrication method. This method is described visually in Figure 2. We chose PDMS as our flexible substrate because of its many attractive properties. PDMS is a biocompatible material with a degree of flexibility suitable for our purposes which grows increasingly robust with increasing thickness. This means that a relatively thin sheet of approximately 250 µm is strong enough to be easily handled and manipulated—an important characteristic for attaching the flexible array to any desired curvilinear surface. 

For the micromachining process, it is necessary to make use of a carrier wafer of a 3-inch glass wafer (after clean room work has been completed, PDMS can simply be peeled off the wafer). PDMS was deposited on the carrier wafer via spin coating at 500 rpms for 30 s. The PDMS was then allowed to self-planarize on a level hot plate before being cured at 75 °C for 7 h. This process yielded a PDMS layer of approximately 250 µm with sufficient adhesion to underlying glass wafer to not cause any further processing difficulties during micromachining. There is, however, one key drawback to the choice of PDMS for our substrate. The low surface energy of PDMS means that metals exhibit very low adhesion to the substrate [11], and even when adhesion difficulties are overcome by applying a plasma treatment to the PDMS prior to metal deposition, the metal thin film remains highly susceptible to propagating spider-web cracking.

In order to overcome this difficulty, we chose to deposit Parylene C as an adhesion layer between PDMS and the copper electrodes of our devices. Parylene C is ideally suited to this task because it produces a conformal coat with outstanding adhesion to cured and untreated PDMS. It additionally serves as a protective barrier for the PDMS in later processing of the substrate—PDMS is prone to swelling when exposed to water and other liquids which are standard to micromachining processes such as lithography, but Parylene C has a very low permeability to moisture. Additionally, when deposited in thin enough layers, Parylene C does not interfere with the flexibility of the substrate as a whole. We therefore deposited a 10 µm layer of Parylene C on our PDMS substrate and subsequently moved on to metal deposition.

Next, a copper thin film of 275 nm was deposited onto the PDMS and Parylene C substrate via magnetron sputtering. Deposition was conducted at the low power of 200 W in order to minimize the heating to the substrate which is inherent in the deposition process and has the potential to induce cracking in the copper thin film. Patterning of the copper was conducted using the standard photolithography processes. Photoresist (S 1813, MicroChem Corp., Westborough, MA, USA) was exposed, developed, hard baked, and then used as a mask for the copper, which was wet etched in a commercial copper etchant (APS-100 copper etchant, Transene Company, Danvers, MA, USA) diluted with DI water in a 4 to 1 ratio (DI water to etchant) at room temperature for 3 min. 

After the photoresist was stripped and the wafer cleaned, a second layer of Parylene C was deposited over the patterned electrodes. This second conformal and pinhole free film of 2.5 µm of Parylene C served as the insulating, dielectric layer to prevent shorts from occurring in our electrowetting devices. This encapsulated the entire wafer in a strong dielectric material (dielectric constant of 3.10 at 1 kHz) with large breakdown voltage (5600 V/mil) [15] in order to ensure robust operation of our electrowetting devices. However, to gain access to the contact pads on the edges of the wafer, where external voltage will be applied to the electrowetting devices, a Parylene C etching step is required. A thick photoresist (AZ P4620, MicroChemicals GmbH, Ulm, Germany) was used to mask the whole wafer except the area directly over the contacting pads, and reactive ion etching (RIE) was used to etch through the Parylene C. The wafer was etched using a 400 W RIE oxygen plasma etch for 4 min.

The final step in the micromachining process was to define a highly hydrophobic circular region immediately over the areal density modulated electrodes. To achieve this hydrophobic region, we used a 1 µm thin film of an amorphous fluoropolymer CYTOP (CTX-809SP2, a 9% solution by weight, AGC Chemicals, Tokyo, Japan) on top of the Parylene C. This multilayer approach further reduces the chances of a short occurring in the device [16], but most importantly defines a hydrophobic region where the oil droplet can be initially placed. This is because the highly hydrophobic surface will prefer silicone oil over water, which should induce pinning at the boundary of the CYTOP disk. To achieve an array of properly patterned disks over our electrode arrays, the first step was to spin coat CYTOP at 4000 rpms. The CYTOP was then cured. Curing temperature was kept as low as feasible because of potential damage to the array from mismatched thermal expansion coefficients between PDMS and Parylene C. The wafer was transferred to an oven were it was prebaked at 50 °C for 1 h before the temperature was gradually increased to 110 °C and held for 6 h before being slowly allowed to cool back to room temperature. Standard curing procedures for CYTOP call for a baking temperature of at least 180 °C, however the maximum temperature of 110 °C was chosen because it slightly exceeds the value of the glass transition temperature of CYTOP, which is 108 °C. Ensuring the temperature was above the glass transition temperature allowed for superior evaporation of solvents from the CYTOP film [17]. After curing, the CYTOP was patterned in a process similar to that used to pattern the Parylene C. Photoresist (S 1813) was used to mask the array of disks and then the CYTOP was etched in a low power oxygen plasma RIE (200 W), which has the additional advantage of acting as a surface treatment for exposed Parylene C to render it temporarily hydrophilic. The photoresist is then carefully stripped, thus completing micromachining.

**Figure 3:**
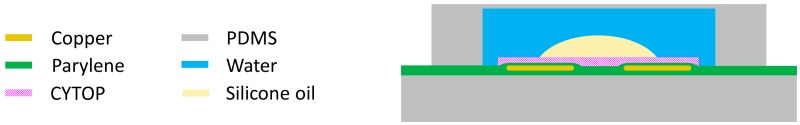
Cross-section of a schematic of a single, fully assembled electrowetting cell. The well for containing the liquids for electrowetting was affixed using PDMS, and a silicone oil droplet was placed over the hydrophobic region of the flexible dielectric surface defined by the patterned CYTOP before being surrounded by water and sealed into the well.

**Figure 4:**
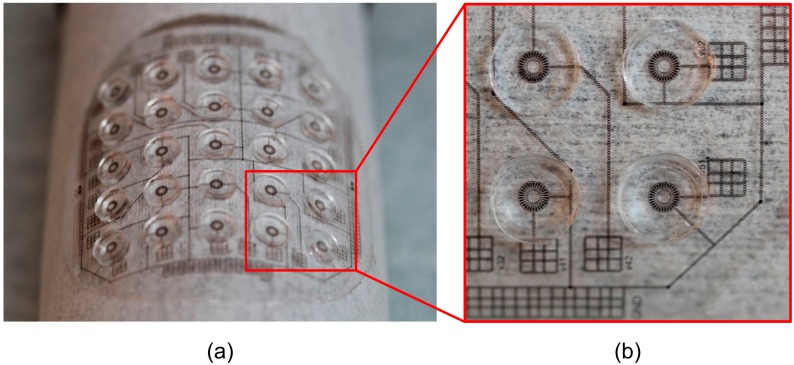
Photographs of the fully assembled flexible electrowetting array on PDMS substrate showing: (**a**) the full 5 × 5 array wrapped on the surface of a cylinder and (**b**), a close up of one section of the array, showing individual electrowetting wells as well as traces and contact pads for applying voltage.

The final step in assembling the array is to attach rings molded from PDMS to the area around each electrode array to form the well to hold the two immiscible liquids. This is done using uncured PDMS as an adhesive between the rings and flexible substrate and manually aligning the rings to the array under the microscope. Once this PDMS adhesive layer has been cured, the PDMS substrate can be carefully peeled off the carrier wafer, and the final step is to fill the wells with water (0.001 M KCl, 150 µS/cm) and silicone oil and then cover them to prevent liquid evaporation. A schematic cross section of a single electrowetting cell can be seen in Figure 3. Images of the fully assembled flexible array can be seen in Figure 4. At this point, the array is ready for voltages to be applied to pads so that electrowetting actuation can be observed. 

## 3. Results

### 3.1. Simulation

Simulations were conducted in order to establish expected actuation of an individual electrowetting cell of the flexible array, as well as verify its mechanical and electrical feasibility. We chose to use ANSYS Fluent to gauge the change in shape of the oil droplet as voltage is applied to the electrowetting actuator. Since the devices were designed to be actuated via electrowetting on a two-layer dielectric the standard electrowetting equations can be applied to correlate voltage applied to the system to the expected contact angle. In the case of an insulator surrounded by a conductive liquid as in our design that equation can be expressed by Equation (2) [18]:

(2) $$\cos\theta =\cos\theta_{0}-\frac{\varepsilon_{0}\varepsilon_{r}}{2d\gamma }V^{2},$$

where $\theta_{0}$ is the initial contact angle of the insulator with external voltage applied, $\varepsilon_{r}$ is the relative permittivity of the dielectric material, $\varepsilon_{0}$ is the permittivity of free space, d is the thickness of the dielectric layer, γ is the interfacial surface tension of the two liquids, V is the voltage applied to the system, and θ is the resulting contact angle of the insulating liquid. 

In our case, the conductive liquid is not intrinsically grounded, but instead allowed to function as a floating electrode. This means we expect to have to apply twice the value of the voltage specified in Equation (2) in order to see the same change in contact angle, θ. Additionally, since our dielectric layer is in fact composed of two separate dielectrics (Parylene C and CYTOP) we can calculate an effective value of $\varepsilon_{r}/d$. Since the dielectrics are of constant thickness and one atop the other they can be considered to be in series so that we can write:

(3) $$\frac{\varepsilon_{r}}{d}=\frac{\varepsilon_{1}}{d_{1}}+\frac{\varepsilon_{2}}{d_{2}},$$

where $\varepsilon_{1}$ and $\varepsilon_{2}$ are the relative permittivities of each of the dielectric layers and $d_{1}$ and $d_{2}$ are their respective thicknesses. 

Because of the highly hydrophobic nature of the CYTOP disk over the areal density modulated electrodes, any oil droplet of reasonable size should spread to cover the whole hydrophobic region to minimize the energy of the system. For the purpose of our simulation, we chose a silicone oil volume of 0.89865 µL. For this volume the initial contact angle of oil correlates to 40°. We chose as an area of interest contact angles between this initial, resting state value and 90°. From Equation (2) we see that this range corresponds to a voltage range of 0 to 71.56 V. 

We set up ANSYS Fluent to run a multiphase model, which allows us to track the interface of two, user defined, immiscible liquids—in our case water and silicone oil with a viscosity of 20 cSt. We specified appropriate densities and viscosities for the liquids in the simulation set up. We also specified the interfacial surface tension coefficient as 24.34 dyn/cm [19]. The geometry of our system was imported to ANSYS and regions of different wettability were identified. The wettability of the CYTOP surface directly over the electrodes of our system was set to vary according to Equation (2), and thus changed from one simulation to the next. The substrate, sidewalls, and lid of our chamber—which corresponded to the edges of our domain in ANSYS—were defined as constantly hydrophilic (contact angle of water equal to 10°), and the regions inside of the CYTOP disk but not directly above an electrode were defined as constantly hydrophobic (contact angle of water equal to 140°). 

**Figure 5:**
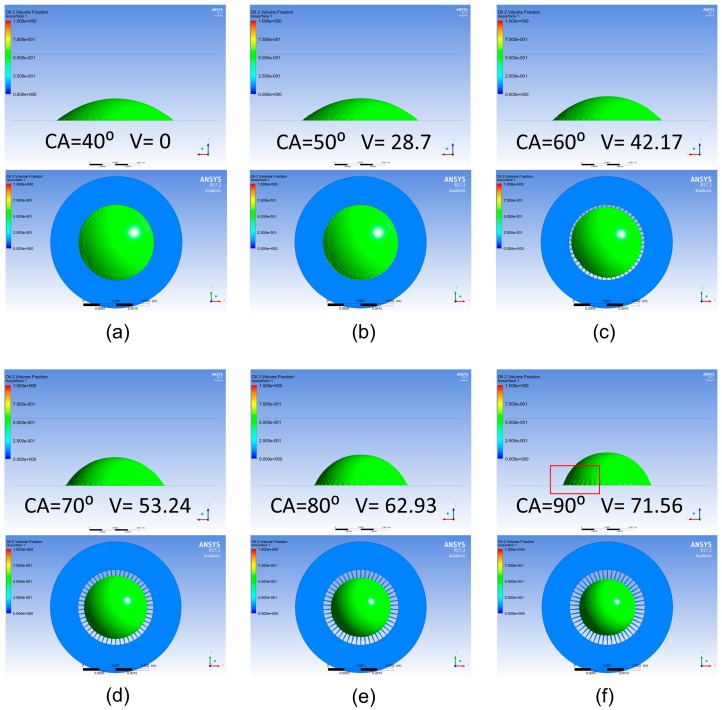
Computational results of ANSYS Fluent simulation of silicone oil droplet surrounded by water under different levels of electrowetting actuation. The underlying electrode structure is shown in blue, the surface of the interface between water and oil is shown in green. Scalloping effect at interface is highlighted by red square. (**a**) The initial state of the fluidics system showing both top and profile view. The 40° contact angle of the oil is equivalent to no externally applied voltage; (**b**) The contact angle changes to 50° at 28.7 V after 100 ms elapsed simulation time; (**c**) the contact angle changes to 60° at 42.17 V after 100 ms; (**d**) the contact angle changes to 70° at 53.24 V after 100 ms; (**e**) the contact angle changes to 80° at 62.93 V after 100 ms; (**f**) the contact angle changes to 90° at 71.56 V after 100 ms.

Simulation solutions were calculated using a non-iterative time step advancing method within the ANSYS software. In order to reduce simulation times, the size of each time step was allowed to vary in accordance with the global courant number of simulation (set to 2.0). To ensure the most accurate interface of our two immiscible liquids, geometric reconstruction was used to evaluate the interface at each step. Each simulation began at the natural state of the system (contact angle of silicone oil equal to 40°) and then saw an abrupt change of surface energies, leading to a corresponding change in contact angle as the simulation progresses. Each simulation was allowed to run for 100 ms in order to allow damping of transient oscillates as the oil droplet moves. The results of these simulations can be seen in Figure 5.

Under close observation, we note a scalloping affect at the edges of the water-silicone oil-CYTOP interface. This is to be expected, since it corresponds to the boundaries of our interdigitated electrodes. The silicone oil directly over the electrodes is squeezed strongly, causing the overall actuation, but in the spaces between electrodes the surface is still highly hydrophobic. This means that the surface prefers the silicone oil to the surrounding liquid and the local contact angle of the silicone oil is less. We also observe that all simulations appeared to have reached a steady state position by the completion of the 100 ms of calculation.

**Figure 6:**
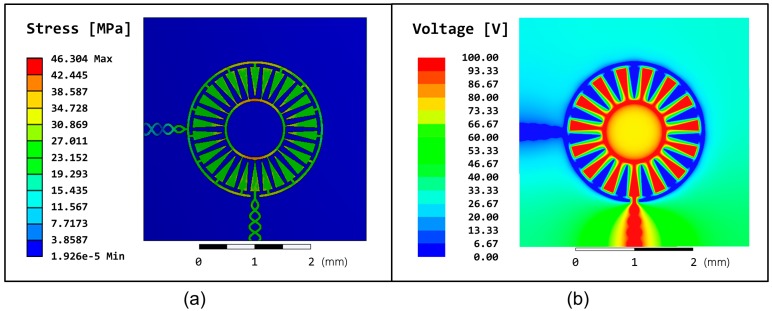
(**a**) Static structural ANSYS simulation results of von Mises equivalent stress in a single electrowetting cell which has been wrapped around a cylinder of radius equal to 30 mm. Maximum stress is observed in the copper electrodes, but at levels well below the fracture point of copper; (**b**) ANSYS Maxwell electrostatic simulation of region on top of Parylene C and CYTOP insulators, where one set of interdigitated electrodes is ground, and the other set has had 100 V applied.

Simulations were also conducted to test the mechanical and electrical integrity of the electrowetting array design. ANSYS was used to create a static structural simulation of a single cell of the array in order to get a measure of the stress (von Mises, equivalent) which results when the substrate is wrapped around a cylinder with a radius of 30 mm (as in Figure 4). The simulation model was constructed to replicate the dimensions of the fabricated device and used the values of Young’s modulus and Poisson’s ratio found in Table 1. The results of this simulation, which show the only significant stress occurring within the copper electrodes, can be seen in Figure 6a. A maximum value of 46.304 MPa is returned for the simulation, with a stress generally at or below 40 MPa for large or crucial regions of the electrode, all of which are well below the fracture strength for copper. 

Finally, an electrostatic simulation was conducted in ANSYS Maxwell to determine the electrical potential on top of the insulators. Voltage was applied across the electrodes and counter-electrodes at 100 V DC, so that the simulation shows a snapshot of the 100 V AC square wave which is applied to the fabricated devices. The results in Figure 6b confirm that the voltage is uniform over the electrode areas.

### 3.2. Experimental Results

Once we completed fabrication of our flexible array of electrowetting cells, we continued on to evaluate observable actuation when voltage was applied to the underlying pair of electrodes. In order to ensure a good electrical contact with the exposed pads at the edges of our flexible substrate, we used conductive silver epoxy to affix wires and more easily apply voltage this way, without fear of damaging the electrical pads via probing. The flexible substrate was laid across an optically transparent, planar support and then place under the microscope. This allowed us to observe the silicone oil–water interface with good clarity.

**Figure 7:**
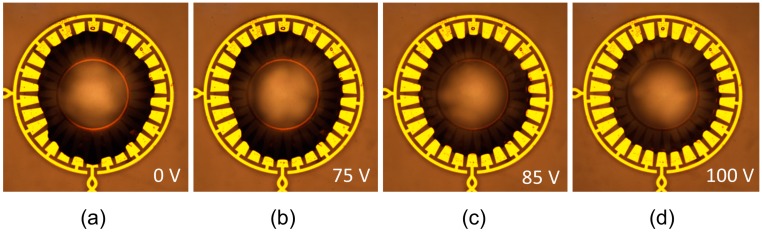
Top view of the silicone oil droplet surrounded by water over electrowetting electrodes. Images were taken via microscope under 5× magnification as voltage (10 kHz, AC square wave) was applied to the system. (**a**) Initial position of the oil droplet, no voltage applied; (**b**) the oil droplet begins to be squeezed by surrounding water at 75 V (RMS); (**c**) droplet when 85 V (RMS) is applied; (**d**) droplet when 100V (RMS) is applied.

As expected, we observed that the oil droplet was squeezed inwards as increasing voltage was applied to the system, because the voltage change attracts the water downwards and inwards to minimize the capacitance of the system. The applied voltage was chosen as an AC square wave with a frequency of 10 kHz, and the resulting actuation can be seen in Figure 7 (all voltages measured as RMS values). It is worthy of note that while the initial placement of the oil droplet over the electrodes was significantly asymmetric, the oil droplet is manipulated into an increasingly circularly symmetric shape as applied voltage is increased. This is as expected for our areal density array design. To minimize the capacitance of the system, the oil droplet will center itself over the electrodes as voltage is applied.

## 4. Discussion and Conclusions

We have demonstrated our fabricated EWOD array is capable of actuation, and that the electrode design does induce a centering of the oil droplet, as expected. Further, we note that the area covered by the oil droplet in our experimental results decreases as increasing voltage is applied. This corresponds to an increase in the contact angle, just as was observed in our simulation results. Therefore, this trend is consistent across simulation and experimental results. Experimental testing required somewhat higher applied voltages then were required to induce actuation than our simulation results may have led us to believe. This discrepancy can be attributed to a number of factors. One possibility is that imperfections in the dielectric layers, particularly in the CYTOP layer since the fluoropolymer is applied via spin coating. These imperfections may effectively lower the relative permittivity of the CYTOP and thus increasing the voltage required for the same actuation. Another possible source of this increased voltage requirement may be charge entrapment in the CYTOP layer [20,21], effectively shielding some of the applied voltage. 

We have also shown the feasibility of fabricating an electrowetting array composed exclusively of flexible materials, allowing it to operate while wrapped on any curvilinear surface. Copper traces designed with flexibility in mind also improve the robustness of flexibility in our design. Robustness of the array was further improved because the conductive liquid in the liquid well for each electrowetting actuator is allowed to float instead of being physically tied to ground of the system, which reduces the chances of electrical shorts. Simulation results demonstrated the effectiveness of symmetric actuation of a droplet of silicone oil to change its contact angle as external voltage is applied to electrodes. In simulation, contact angles ranging from 40° to 90° where observed, corresponding with voltages from 0 to 71.56 V. This same symmetrical squeezing of the oil droplet experiencing electrowetting actuation was observed when fabricated samples were tested. These validate the possibility of future work in applying the basic material and fabrication design to more targeted applications, such as cell culturing and electrowetting lab-on-a-chip applications.

Future work may also pursue the use of a similar design for electrowetting microlens array applications. This work would involve characterizing the optical properties of the silicone oil droplet in water and assessment of aberrations. Work can also be done to reduce the driving voltage, potentially by decreasing dielectric thickness. 

## Figures and Tables

**Table 1:** Values used in static structural ANSYS simulation to determine von Mises equivalent stress.

Materials	Young’s Modulus (MPa)	Poisson’s Ratio
**Copper**	1.1 × 10^5^	0.343
**CYTOP**	1500	0.42
**Parylene C**	2758	0.4
**PDMS**	0.5	0.48

## References

[1] Shields C.W., Reyes C.D., López G.P. (2015). Microfluidic cell sorting: A review of the advances in the separation of cells from debulking to rare cell isolation. Lab Chip.

[2] Pollack M.G., Shenderov A.D., Fair R.B. (2002). Electrowetting-based actuation of droplets for integrated microfluidics. Lab Chip.

[3] Paik P., Pamula V.K., Pollack M.G., Fair R.B. (2003). Electrowetting-based droplet mixers for microfluidic systems. Lab Chip.

[4] Cho S.K., Moon H., Kim C. (2003). Creating, Transporting, Cutting, and Merging Liquid Droplets by Electrowetting-Based Actuation for Digital Microfluidic Circuits. J. Microelectromech. Syst..

[5] Jones T.B., Fowler J.D., Chang Y.S., Kim C.J. (2003). Frequency-based relationship of electrowetting and dielectrophoretic liquid microactuation. Langmuir.

[6] Li C., Jiang H. (2012). Electrowetting-driven variable-focus microlens on flexible surfaces. Appl. Phys. Lett..

[7] Fan S., Huang P., Wang T., Peng Y. (2008). Cross-scale electric manipulations of cells and droplets by frequency-modulated dielectrophoresis and electrowetting. Lab Chip.

[8] Wang H., Dembo M., Wang Y. (2000). Substrate flexibility regulates growth and apoptosis of normal but not transformed cells. Am. J. Physiol. Cell Physiol..

[9] Steckl A.J., You H., Kim D. (2011). Flexible electrowetting and electrowetting on flexible substrates. Proc. SPIE.

[10] Fan S., Yang H., Hsu W. (2011). Droplet-on-a-wristband : Chip-to-chip digital microfluidic interfaces between replaceable and flexible electrowetting modules. Lab Chip.

[11] Lu Y.S., Tu H., Xu Y., Jiang H. (2013). Tunable dielectric liquid lens on flexible substrate. Appl. Phys. Lett..

[12] Murade C.U., Oh J.M., van den Ende D., Mugele F. (2011). Electrowetting driven optical switch and tunable aperture. Opt. Express.

[13] Ren H., Xu S., Ren D., Wu S.-T. (2011). Novel optical switch with a reconfigurable dielectric liquid droplet. Opt. Express.

[14] Ashtiani A.O., Jiang H. (2016). Design and fabrication of an electrohydrodynamically actuated microlens with areal density modulated electrodes. J. Micromech. Microeng..

[15] Li C., Jiang H. (2014). Fabrication and characterization of flexible electrowetting on dielectrics (EWOD) microlens. Micromachines.

[16] Schultz A., Chevalliot S., Kuiper S., Heikenfeld J. (2013). Detailed analysis of defect reduction in electrowetting dielectrics through a two-layer “barrier” approach. Thin Solid Films.

[17] Asiri H. (2012). Fabrication of Surface Plasmon Biosensors in CYTOP.

[18] Liu C., Park J., Choi J. (2008). A planar lens based on the electrowetting of two immiscible liquids. J. Micromech. Microeng..

[19] Than P., Preziosi L., Joseph D.D., Arney M. (1988). Measurement of Interfacial Tension between Immiscible Liquids with the Spinning Rod Tensiometer. J. Colloid Interface Sci..

[20] Khodayari M., Carballo J., Crane N.B. (2012). A material system for reliable low voltage anodic electrowetting. Mater. Lett..

[21] Berry S., Kedzierski J., Abedian B. (2007). Irreversible Electrowetting on Thin Fluropolymer Films. Langmuir.

